# Internet information on birth options after caesarean compared to the RCOG patient information leaflet; a web survey

**DOI:** 10.1186/1471-2393-14-361

**Published:** 2014-10-11

**Authors:** Natalie Whitelaw, Siladitya Bhattacharya, David McLernon, Mairead Black

**Affiliations:** Aberdeen Maternity Hospital, Cornhill Road, Aberdeen, AB25 2ZD UK; Division of Applied Health Sciences, School of Medicine and Dentistry, Aberdeen Maternity Hospital, Cornhill Road, Aberdeen, AB25 2ZD UK; Department of Medical Statistics, Division of Applied Health Sciences, School of Medicine and Dentistry, University of Aberdeen, Polwarth Building, Cornhill Road, Aberdeen, AB25 2ZD UK; Division of Applied Health Sciences, School of Medicine and Dentistry, University of Aberdeen, Aberdeen Maternity Hospital, Cornhill Road, Aberdeen, AB25 2ZD UK

**Keywords:** Caesarean section, Vaginal birth, Internet information

## Abstract

**Background:**

Repeat caesarean sections make a substantial contribution to the overall caesarean section rate. It is important to understand what influences women to choose this option when the alternative of attempting vaginal birth after caesarean section is available. As many such women use the internet while seeking information on their options, the aim of this study was to assess content of websites on birth after previous caesarean and identify website characteristics which predict content.

**Methods:**

An internet survey of the forty eight most frequently encountered websites retrieved from a search using various terms relating to birth after caesarean section via a popular search engine was performed. Websites were assessed for their content supportive of either vaginal birth after caesarean (VBAC) or elective repeat caesarean section (ERCS), using the RCOG patient information document, ‘Birth after previous caesarean; Information for You’ as a ‘gold standard’. A simple scoring method which categorised information into either supportive of VBAC (14 facts available) or ERCS (10 facts available) was employed and mean scores compared. Poisson regression analysis was used to assess the extent to which the score was predicted by website funding source, country of origin, author status and intended audience.

**Results:**

A mean of 42.4% (SD 23.8) of facts supportive of VBAC and 44.8% (SD 25.0) of facts supportive of ERCS were featured across the 48 websites, with corresponding scores in the five most frequently encountered websites being 40.0% (SD 13.9) and 66.0% (SD 20.7). Extent of featured information supportive of ERCS was related to country of origin with the UK having higher scores on average than the US.

**Conclusions:**

Women searching for internet information on birth after previous caesarean are exposed to incomplete information. Origin of website has a significant effect on website content.

## Background

Caesarean sections account for 23.8% of all singleton births within the United Kingdom [1]. Elective repeat caesarean section (ERCS) is currently the most substantial primary indication for caesarean section (CS) [2], although planned vaginal birth after caesarean (VBAC) is an acknowledged acceptable alternative [3–5]. Within contemporary health services striving to deliver patient-centred care, women with a history of previous CS should be allowed to attempt to achieve the mode of delivery which they perceive to offer the greatest benefit to them and their offspring [6]. In order to ensure informed decision making in this group, information must be provided, but evidence suggests that this is currently lacking and that women seek additional information from various sources including the internet [7].

Although there are currently no randomised controlled trials which compare the risks and benefits of VBAC versus ERCS [2], the Royal College of Obstetricians and Gynaecologists (RCOG) guidelines and audit committee have compiled a patient information leaflet on birth after previous caesarean section using the best available scientific evidence at that time [8]. This document complements the RCOG clinical guidance developed to assist health professionals and pregnant women in choosing either VBAC or ERCS, which includes precise risks of each delivery option for discussion [3].

It is known that most women have already formed an opinion on preferred delivery type after previous caesarean before attending antenatal care in their next pregnancy [9, 10]. The internet is an important source of information in this context [11, 12]. It is currently unknown whether the most widely accessible website information on birth after previous caesarean section is evidence-based or non-biased, and therefore reflective of the information contained within the RCOG patient information document. Although a number of tools exist in order to assess internet related healthcare information [13–16], the quality of such information is often poor and assessment by the general public sub-optimal [17]. Considering health professionals’ responsibility to ensure that women make informed health-related decisions, an appreciation of the quality of internet based healthcare information accessed by their patients is imperative.

The aim of this survey was to assess completeness of data provided by the most frequently encountered websites during a search for information on mode of delivery after caesarean section, and to compare the extent to which information is featured in support of ERCS with that in support of VBAC. Additionally, it was intended that website characteristics which predict completeness of information in support of either mode of delivery be identified.

## Methods

### Internet search method

The most popular search engine worldwide, Google™ (https://www.google.com)[18], was used to perform the internet search. A single reviewer performed a pilot search using the pre-defined search term outlined in Table 1. The first ten web links identified were used to test the simple scoring system devised by the authors at the study outset. A combination of ten further search phrases were performed, which the authors believed would closely resemble terms that lay searchers would utilise to reach relevant websites providing information on birth after caesarean section (Table 1). Where a number of synonyms were possible (i.e. delivery, pregnancy, birth), an additional search was performed using Boolean operators to increase the relevant yield. These searches were performed in March 2012.

**Table 1:** **Search phrases utilised**

Search number	Search term
Pilot search	Birth after previous caesarean birth
1	Next (Delivery, pregnancy, labour) Birth after (previous) caesarean birth (section, delivery, c-section)
2	(Elective) repeat caesarean birth (section, delivery, c-section)
3	Normal (natural, vaginal) birth (delivery) after caesarean birth (section, delivery, c-section)
4	Previous caesarean birth (section/delivery, c-section)
5	VBAC
6	ERCS
7	Vaginal (natural, normal, VBAC) delivery (birth, labour) or repeat caesarean birth (section/delivery, c-section, ERCS) after previous caesarean birth (section/delivery, c-section)
8	(Type, mode) of delivery after caesarean birth (section/delivery, c-section)
9	(Advantages and disadvantages (risks and benefits) of) type of delivery (birth, labour) for next birth (pregnancy, labour, birth) after caesarean birth (section/delivery, c-section)
10	Options for (next) delivery (birth, labour, pregnancy) after caesarean birth (section/delivery, c-section)

**Figure 1:**
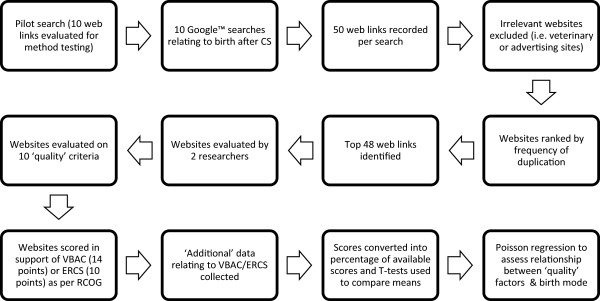
**Search process.**

Initially, the 50 websites most frequently returned using each of the searches were recorded. This number was chosen as most lay searchers do not look beyond the first page of results and concordance between common search engines is approximately 50% [17, 19]. Website links were excluded if they did not appear to contain any information relevant to the key terms entered, in either the link itself or the surrounding text (for example veterinary, advertising or dictionary websites). The search process is outlined in Figure 1.

### Criteria for evaluating websites

Each website was accessed between March 2012 and April 2013 by two reviewers independently and data collected by accessing the relevant pages of the website. Once accessed, all websites were included whether felt to be relevant to birth after previous caesarean section or not.

The data collected for each website included ten generally agreed key characteristics to help assess quality and relevance of health information on the internet: Authority of source; Purpose of website; Date of last update; Country of origin; Intended audience; Provision of disclaimer; Funding source; Contact details; References; Links [13, 14, 16, 17]. If websites mentioned any of the advantages and disadvantages of VBAC or ERCS in the RCOG patient information leaflet on birth after previous caesarean, these were recorded and scored [8]. If additional advantages or disadvantages of either mode of delivery were mentioned on the website this was also recorded and categorised into ‘supporting VBAC’ or ‘supporting ERCS’, but not included in the score.

### Analytic methods

A simple scoring system for each website was devised by the authors at the outset of the study. This consisted of 10 points available for website ‘quality’ features and 24 points taken from the gold standard RCOG patient information. The latter was further subdivided into 14 potential points which support VBAC and 10 potential points which support ERCS. These subdivisions involved criteria which convey advantages of the mode of delivery in question and disadvantages of the alternative mode of delivery (Table 2). Any disparities in scores obtained by the two reviewers were dealt with by further assessment together in order to reach agreement on a final score. The ERCS and VBAC scores were calculated as a percentage of the total available score in order to compare the level of support for each mode of delivery within each site. A paired t-test was used to test whether there was a significant difference between the two mean percentage scores for ERCS and VBAC. The scores of the top five most frequently returned websites were also calculated. The pragmatic choice to consider the top five websites as a subgroup was made because these websites are potentially the most frequently accessed and may be accessed by women who dedicated little time to their search.

**Table 2:** **Number of websites addressing delivery mode advantages and risks mentioned in RCOG patient information document ‘Birth after previous caesarean: Information for you’**

Mode of delivery supported		Individual points available for advantages or risks	Number of websites addressing criterion (n = 48)
**In support of VBAC (14 criteria)**	**VBAC advantages (7 criteria)**	Experiencing a vaginal birth	15
		Greater chance of uncomplicated birth in future	25
		Shorter recovery	28
		Shorter hospital stay	28
		Less abdominal pain	14
		Not having surgery	23
		Reduced risk of neonatal respiratory morbidity	12
	**ERCS risks (7 criteria)**	Longer operation	4
		Difficult operation	7
		Risk of thrombosis	26
		Longer recovery	22
		Neonatal breathing problems	30
		Need for ERCS in future	14
		Increased complications with each ERCS	16
**In support of ERCS (10 criteria)**	**VBAC risks (6 criteria)**	Emergency c-section	27
		Blood transfusion	14
		Endometritis	24
		Uterine scar rupture	45
		Perinatal death	30
		Neonatal brain damage	22
	**ERCS advantages (4 criteria)**	Less risk of uterine rupture	21
		Less risk of stillbirth	10
		Less risk neonatal brain damage	12
		Known delivery date	10

Poisson regression analysis was used to assess the effect of website quality characteristics on scores achieved in support of each mode of delivery. To adjust for over-dispersion, the Pearson chi-square method was used to estimate the scale parameter. As an extra check, we also fitted a negative binomial model and used the Lagrange multiplier test to test whether the ancillary parameter equalled zero. A non-significant result meant that over-dispersion was not a concern. The website characteristics which were considered included: authority of source (‘included health professional’ or ‘did not include health professional’); country of origin (‘UK’, ‘US’ or ‘other’); intended audience (‘included the general public’ or ‘included health professionals’) and funding source (‘commercial’, ‘government’ or ‘other’). These characteristics were selected as they were considered likely to influence whether or not women would consult a particular website. For example, some women might seek websites where information is relevant to their country (and therefore healthcare system), while others are more trusting of government (e.g. NHS) websites. Statistical analyses were performed using Statistical Package for Social Scientists Version 18.

## Results

### Pilot search

The pilot search produced quality scores ranging from 8 to 10 out of a possible 10, with seven sites scoring for all 10 parameters. The content scoring revealed that each piece of information suggested in the RCOG document featured on at least two websites and at least two pieces of information featured on all ten websites. The search strategy was not altered following the pilot search. The pilot results were not included in the final set of results.

#### Main data

##### Search results

 A total of 950 web links were returned using the search terms outlined in Table 1. After excluding duplicate web links, a total of 298 individual websites were counted. Of these, 48 were counted five times or more and were included for analysis (Table 3). Exactly 50 websites could not be distinguished using the chosen counting process, as 13 sites appeared four times overall. The most popular website appeared 27 times across the various searches, reflecting the repetitive nature of links appearing on any one list of search results.

**Table 3:** **Top 48 websites returned**

Website rank (count frequency)	Website	Country of origin	Funding source	Authority of source	Intended audience	Date last accessed	Website quality characteristic score (maximum score = 10)	VBAC score (% available score)	ERCS score (% available score)
1	Wikipedia. Vaginal birth after caesarean. 2013; http://en.wikipedia.org/wiki/Vaginal_birth_after_caesarean.	USA	Other	Other	General public/parents	24^th^ August 2013	10	35.7	50.0
2	Babycenter medical advisory board. Vaginal birth after caesarean (VBAC). http://www.babycenter.com/0_vaginal-birth-after-cesarean-vbac_1420895.bc.	UK	Commercial	Health Professionals	General public/parents	24^th^ August 2013	8	35.7	70.0
3	Childbirth connection. VBAC or repeat C-section. 2012; http://www.childbirthconnection.org/article.asp?ck=10210.	USA	Other	Other	General public/parents	24^th^ August 2013	9	35.7	50.0
4	BabyCentre medical advisory board. Vaginal birth after caesarean (VBAC). 2013; http://www.babycentre.co.uk/a557727/vaginal-birth-after-caesarean-vbac.	UK	Commercial	Health professionals	General public/parents	24^th^ August 2013	10	64.3	100.0
5	Martel M, MacKinnon C. Guidelines for Vaginal Birth After Previous Caesarean Birth. 2005; http://www.sogc.org/guidelines/public/155E-CPG-February2005.pdf.	Other	Other	Health professionals	Includes Professionals	24^th^ August 2013	8	28.6	60.0
6	Wells C, Cunningham F. Choosing the route of delivery after cesarean birth. 2013; http://www.uptodate.com/contents/choosing-the-route-of-delivery-after-cesarean-birth.	USA	Commercial	Health professionals	Includes Professionals	24^th^ August 2013	10	42.9	70.0
7	Healthwise Staff. Vaginal Birth After Caesarean (VBAC). 2011; http://www.healthlinkbc.ca/kb/content/special/hw200557.html.	Other	Government	Health professionals	General public/parents	24^th^ August 2013	10	64.3	50.0
8	Caughey A. Vaginal Birth After Cesarean Delivery. 2011; http://emedicine.medscape.com/article/272187-overview.	USA	Commercial	Health professionals	Includes Professionals	24^th^ August 2013	10	21.4	50.0
9	Royal College of Obstetricians and Gynaecologists. Birth after previous caesarean - information for you. 2008; https://www.rcog.org.uk/en/patients/patient-leaflets/birth-after-previous-caesarean/.	UK	Other	Health professionals	Includes Professionals	24^th^ August 2013	7	100.0	100.0
10	Ben-Joseph E. Can I Have a Vaginal Birth If I Had a Previous C-Section? 2012; http://kidshealth.org/parent/question/infants/vbac.html#cat20730.	USA	Other	Health professionals	General public/parents	24^th^ August 2013	9	42.9	10.0
11	American Pregnancy Association. VBAC: Vaginal Birth after Cesarean. 2012. http://americanpregnancy.org/labornbirth/vbac.html.	USA	Commercial	Health professionals	General public/parents	24^th^ August 2013	8	42.9	10.0
12	Joy S, Contag S. Cesarean Delivery. 2013; http://emedicine.medscape.com/article/263424-overview.	USA	Commercial	Health professionals	Includes Professionals	24^th^ August 2013	10	35.7	50.0
13	MedicineNet. Cesarean Birth (C-Section). http://www.medicinenet.com/c-section_cesarean_birth/article.htm.	USA	Commercial	Health professionals	General public/parents	24^th^ August 2013	8	50.0	20.0
14	Sehdev H. Vaginal Birth After Cesarean Delivery. http://www.emedicinehealth.com/vaginal_birth_after_cesarean_delivery/article_em.htm.	USA	Commercial	Health professionals	General public/parents	24^th^ August 2013	10	64.3	70.0
15	Dodd J, Crowther C, Hiller J, Haslam R, Robinson J. Birth after caesarean study – planned vaginal birth or planned elective repeat caesarean for women at term with a single previous caesarean birth: protocol for a patient preference study and randomised trial. 2007; http://www.biomedcentral.com/1471-2393/7/17.	Other	Other	Other	Includes Professionals	24^th^ August 2013	8	28.6	80.0
16	Mayo Clinic Staff. Vaginal birth after cesarean (VBAC). 2012; http://www.mayoclinic.com/health/vbac/MY01143.	USA	Other	Health professionals	General public/parents	24^th^ August 2013	10	64.3	50.0
17	WebMD. Vaginal Birth After C-Section (VBAC) Directory. http://www.webmd.com/baby/vaginal-birth-after-section-vbac-directory.	USA	Commercial	Health professionals	General public/parents	24^th^ August 2013	10	64.3	50.0
18	Weiss R. Cesarean Section Photos: Step-by-Step. http://pregnancy.about.com/od/cesareansection/ss/cesarean.htm.	USA	Commercial	Other	General public/parents	24^th^ August 2013	7	71.4	40.0
19	BabyCenter. Vaginal birth after caesarean (VBAC). 2010; http://www.babycenter.ca/pregnancy/labourandbirth/labourcomplications/vbac/.	Other	Commercial	Health professionals	General public/parents	24^th^ August 2013	9	42.9	40.0
20	Bowen M. Caesarean section. http://www.netdoctor.co.uk/health_advice/facts/caesarian.htm.	UK	Commercial	Health professionals	General public/parents	24^th^ August 2013	9	21.4	20.0
21	Kamel J. VBAC Facts. 2012; http://vbacfacts.com.	USA	Commercial	Other	General public/parents	24^th^ August 2013	9	21.4	80.0
22	March of Dimes. Vaginal birth after cesarean. 2011; http://www.marchofdimes.com/pregnancy/vaginal-birth-after-cesarean.aspx.	USA	Other	Health professionals	General public/parents	24^th^ August 2013	9	50.0	30.0
23	Ourbodies OS. Vaginal Birth After Cesarean (VBAC) or Repeat Cesarean Section? 2005; http://www.ourbodiesourselves.org/book/childbirthexcerpt.asp?id=85&chapterID=21.	USA	Other	Other	General public/parents	24^th^ August 2013	7	71.4	30.0
24	Women’s and Children’s Health Network. Next birth after caesarean section. 2008; http://www.cyh.com/HealthTopics/HealthTopicDetails.aspx?p=438&np=463&id=2824.	Other	Government	Health professionals	General public/parents	24^th^ August 2013	10	85.7	40.0
25	Ask Dr Sears. Vaginal Birth After Cesarean. http://www.askdrsears.com/topics/pregnancy-childbirth/pregnancy-concerns/vaginal-birth-after-cesarean.	USA	Other	Health professionals	General public/parents	24^th^ August 2013	6	7.1	20.0
26	BabyZone, Disney. VBAC. http://www.babyzone.com/pregnancy/labor-and-delivery/vbac/.	USA	Other	Health professionals	General public/parents	24^th^ August 2013	6	21.4	70.0
27	Guise JM, Eden K, Emeis C, Denman MA, Marshall N, Fu RR, Janik R, Nygren P, Walker M, McDonagh M. Vaginal birth after cesarean: new insights. 2010; http://www.ncbi.nlm.nih.gov/pubmed/20629481.	USA	Commercial	Health Professionals	Includes professionals	24^th^ August 2013	6	7.1	30.0
28	Kings College Hospital NHS Foundation Trust. Birth after caesarean section; Information for women. 2010; https://www.kch.nhs.uk/Doc/pl%20-%20221.1%20-%20vaginal%20birth%20after%20caesarean%20section.pdf.	UK	Government	Health professionals	General public/parents	24^th^ August 2013	7	64.3	30.0
29	Murkoff H. VBAC - or Not VBAC. http://www.whattoexpect.com/pregnancy/labor-and-delivery/cesarean-section/vaginal-birth-after-c-section.aspx.	USA	Commercial	Health professionals	General public/parents	24^th^ August 2013	8	28.6	10.0
30	North Lincolnshire and Goole Hospitals NHS Foundation Trust. Vaginal birth after previous caesarean section (VBAC). 2011; http://www.nlg.nhs.uk/content/uploads/2013/12/IFP-0075VaginalBirthAfterCsection.pdf.	UK	Government	Health professionals	General public/parents	24^th^ August 2013	9	85.7	80.0
31	Skelton P. Vaginal birth after caesarean – VBAC. http://www.kiwifamilies.co.nz/articles/vaginal-birth-after-caesarean-vbac/.	Other	Commercial	Health professionals	General public/parents	24^th^ August 2013	7	42.9	20.0
32	TAJ G, SOHAIL N, CHEEMA S, ZAHID N, RIZWAN S. Review of Study of Vaginal Birth After Caesarean Section (VBAC). 2008; http://www.annalskemu.org/journal/index.php/annals/article/view/102/90.	Other	Other	Health Professionals	Includes professionals	24^th^ August 2013	6	0.0	20.0
33	Tita AT, Landon MB, Spong CY, Lai Y, Leveno KJ, Varner MW, Moawad AH, Caritis SN, Meis PJ, Wapner RJ, Sorokin Y, Miodovnik M, Carpenter M, Peaceman AM, O’Sullivan MJ, Sibai BM, Langer O, Thorp JM, Ramin SM, Mercer BM, Eunice Kennedy Shriver NICHD Maternal-Fetal Medicine Units Network. Timing of elective repeat cesarean delivery at term and neonatal outcomes. 2009; http://www.ncbi.nlm.nih.gov/pubmed/19129525.	USA	Other	Health Professionals	Includes professionals	24^th^ August 2013	8	7.1	00.0
34	Wrightington, Wigan and Leigh NHS Foundation Trust. Vaginal birth following caesarean section; patient information. 2012; https://www.google.com/url?q=http://www.wwl.nhs.uk/library/all_new_pi_docs/audio_leaflets/obstetrics/csection/vbac.pdf&sa=U&ei=GbdGVKjRJdLd7Qad7YCYDA&ved=0CAUQFjAA&client=internal-uds-cse&usg=AFQjCNHQIZ0H1jlK_UORGNpVKHWArjhtaQ.	UK	Government	Health professionals	General public/parents	24^th^ August 2013	7	21.4	60.0
35	Trial of labor after cesarean (TOLAC). http://www.aafp.org/online/etc./medialib/aafp_org/documents/clinical/patient_ed/tolac-color.Par.0001.File.tmp/TOLAC-color.pdf.	USA	Government	Health professionals	General public/parents	24^th^ August 2013	7	35.7	40.0
36	American Baby. Vaginal birth after cesarean. http://health.howstuffworks.com/pregnancy-and-parenting/pregnancy/labor-delivery/vaginal-birth-after-cesarean.htm.	USA	Commercial	Other	General public/parents	24^th^ August 2013	6	28.6	20.0
37	BJOG. Risk of uterine rupture after previous caesarean section. 2010; http://www.bjog.org/details/news/591673/Risk_of_uterine_rupture_after_previous_caesarean_section.html.	UK	Other	Health professionals	Includes professionals	24^th^ August 2013	7	0.0	40.0
38	Crowther C, Dodd J, Hiller J, Haslam R, Robinson J. Planned Vaginal Birth or Elective Repeat Caesarean: Patient Preference Restricted Cohort with Nested Randomised Trial. 2012; http://www.plosmedicine.org/article/info%3Adoi%2F10.1371%2Fjournal.pmed.1001192.	Other	Other	Health professionals	Includes professionals	24^th^ August 2013	9	21.4	80.0
39	Ontario Midwives. Vaginal birth after cesarean (VBAC). http://www.ontariomidwives.ca/care/birth/vbac.	Other	Other	Health professionals	Includes professionals	24^th^ August 2013	6	57.1	50.0
40	Pregnancy Info. Vaginal birth after a cesarean section. http://www.pregnancy-info.net/vaginal_birth.html.	USA	Commercial	Other	General public/parents	24^th^ August 2013	6	64.3	10.0
41	The Newcastle upon Tyne Hospitals NHS FoundationTrust. Choices for birth after a caesarean section. http://www.newcastle-hospitals.org.uk/services/maternity-unit_treatment-and-medication_choices-for-birth-after-a-caesarean-section.aspx.	UK	Government	Health professionals	General public/parents	24^th^ August 2013	8	42.9	50.0
42	The Pregnancy Zone. Vaginal birth after cesarean. 2012; http://www.thepregnancyzone.com/labor-delivery/vaginal-birth-after-cesarean/.	Other	Commercial	Other	General public/parents	24^th^ August 2013	7	64.3	50.0
43	University of Maryland Medical Center. Vaginal Birth after C-Section (VBAC). 2013; http://umm.edu/health/medical/pregnancy/labor-and-delivery/vaginal-birth-after-csection-vbac.	USA	Commercial	Health professionals	General public/parents	24^th^ August 2013	7	28.6	50.0
44	Haas A. Homebirth after Cesarean: The Myth and the Reality. 2008; http://www.midwiferytoday.com/articles/homebirthaftercesarean.asp.	USA	Commercial	Other	General public/parents	24^th^ August 2013	7	35.7	0.0
45	Harrington K. Pregnancy/Labour and delivery. http://www.wellbeingforwomen.com/index.php/pregnancy_topics/labour-and-delivery/#caesarean_section.	UK	Other	Health professionals	General public/parents	24^th^ August 2013	7	7.1	80.0
46	Jukelevics N. VBAC.Com. http://www.vbac.com/.	USA	Other	Other	Includes professionals	24^th^ August 2013	9	42.9	30.0
47	King Edward Memorial Hospital. Vaginal Birth After Caesarean. http://www.kemh.health.wa.gov.au/health/VBAC/index.htm.	Other	Government	Health professionals	General public/parents	24^th^ August 2013	8	71.4	40.0
48	Pregnancy Weekly. Vaginal birth after cesarean (VBAC). http://www.parentingweekly.com/pregnancy/delivery-options/vaginal-birth-after-cesarean_2.htm.	Other	Commercial	Other	public/parents	24^th^ August 2013	5	64.3	50.0

##### Quality assessment of top websites

**Figure 2:**
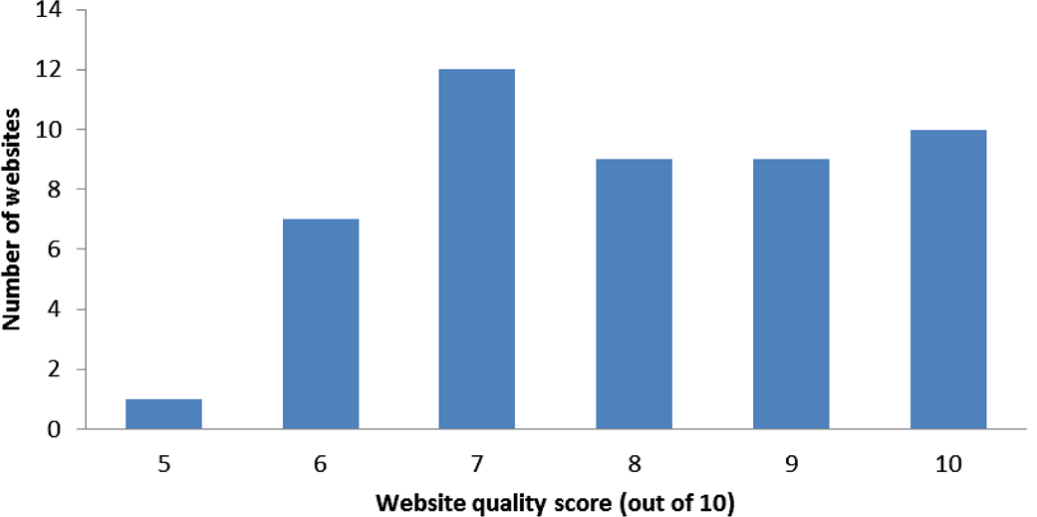
**Website quality characteristic scores.**

 The number of websites displaying the various website ‘quality’ characteristics is illustrated in Figure 2. Essentially, the majority of websites were clearly accredited by health professionals (n = 37), with most aimed at providing information to prospective parents (n = 38). All websites had an available feedback mechanism and displayed country of source. Most websites were sourced from the USA (n = 26), with the main funding source overall being advertising (n = 20). For these ‘quality’ characteristics, each website was given a score out of 10. Less than one quarter (n = 10) of websites scored 10, but all websites scored 5 or more.

##### Comparison of website clinical data to gold standard

**Figure 3:**
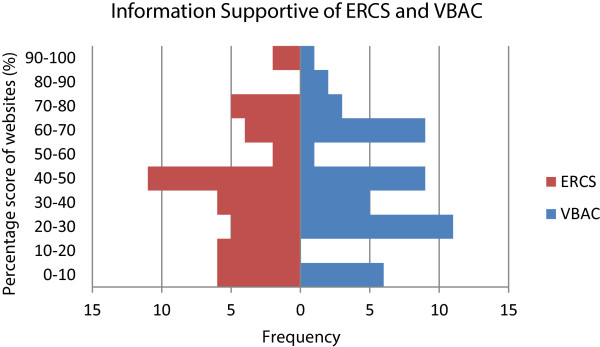
**Website content as a percentage of potential pieces of information in support of each mode of delivery after caesarean birth compared with RCOG patient information document ‘Birth after previous caesarean: Information for you’.**

The number of websites demonstrating individual criteria supporting either VBAC or ERCS as outlined in the RCOG patient document was recorded (Table 2). The percentage score achieved in support of either VBAC or ERCS for each website was calculated (Figure 3). Only one of 48 websites mentioned 100% of points in favour of VBAC as mentioned in RCOG ‘gold standard’ patient information, with just two of 48 websites mentioning 100% of points in favour of ERCS. The most commonly addressed criterion was the increased risk of uterine scar rupture with VBAC (n = 45). Forty sites provided additional information in support of VBAC which not mentioned in the RCOG guidance. Most commonly cited examples included risks of wound infection (n = 21), haemorrhage (n = 18) and maternal surgical injury (n = 14) with ERCS. Twenty seven sites provided additional information in support of ERCS. Most commonly cited examples included the risk of incontinence (n = 7) and traumatic perineal injury (n = 8) following VBAC. Many of these ‘additional’ information points collected were discordant with best available evidence [2, 3].

Of 24 possible pieces of information provided in support of either ERCS or VBAC, the mean number provided by all 48 websites assessed was 10.4 (SD 4.6). As a percentage of the total available points in support of VBAC, the overall mean score was 42.4% (SD 23.8). As a percentage of the total available points in support of ERCS, the overall mean score was 44.8% (SD 25.0). This difference in means was not statistically significant (p = 0.57). Of the five most frequently returned websites, the mean score in support of VBAC was 40.0% (SD 13.9) and in support of ERCS was 66.0% (SD 20.7). Uterine scar rupture (91.7%) with VBAC and increasing complications with each CS (72.9%) were the most frequently mentioned criteria by websites overall, with few websites mentioning that ERCS was a longer (8.3%), more difficult procedure (12.5%).

##### Website characteristics as predictor of content scores

The authority of source, intended audience and funder of the websites did not have any association with the number of supportive criteria presented for either VBAC or ERCS. However, country of origin appeared to impact ERCS total scores, with UK websites reporting 88% higher scores (95% CI 24–186) than USA websites (Table 4). Country of origin had no association with the score for VBAC. For both models the Lagrange multiplier test was not significant meaning that over-dispersion may not be a concern.

**Table 4:** **Effect of website characteristics on the number of supportive criterion met by the website for VBAC and ERCS**

Website characteristic	VBAC (rate ratio with 95% CI)	ERCS (rate ratio with 95% CI)
Funder (versus Commercial)		
Government	1.46 (0.87-2.45)	0.91 (0.55-1.51)
Other	0.89 (0.60-1.32)	1.02 (0.71 -1.49)
Country of origin (versus US)		
UK	0.96 (0.58-1.57)	1.88 (1.24-2.86)
Other	1.13 (0.74-1.73)	1.34 (0.90-2.00)
Authority of source (versus health professional)		
Other	1.21 (0.81-1.80)	1.09 (0.73-1.63)
Intended audience (versus General public)		
Including health professional	0.74 (0.47-1.18)	1.17 (0.78-1.75)

Note: Each column represents one Poisson regression model adjusted for all four website characteristics.

## Discussion

### Main findings

This study has revealed that the content of readily accessed websites with information on birth options after CS reflect that contained in the RCOG patient information document ‘Birth after previous caesarean; information for you’ to a variable extent. At the extreme ends of the spectrum; from 24 key pieces of information which the RCOG recommend women are made aware of; the risk of a long operation with ERCS is discussed in only four of 48 websites assessed while uterine rupture is discussed on 45 of the 48. Less than half of the facts in support of either mode of delivery were featured on all 48 websites. Only country of origin was associated with the number of criteria supportive of ERCS with the UK reporting more supportive criterion than the US. The five most frequently returned websites contained two thirds of the facts supportive of ERCS, and less than half of those supportive of VBAC.

### Strengths and limitations

The initial website searches performed on Google™ were not limited to those in the UK as it was felt unlikely the lay searcher would perform this function. As a result, more than half of the top websites originated from the USA and only 11 from the UK. For this reason, website information could have been skewed towards guidance on birth after caesarean which has been published internationally. On the other hand, no significant differences have been found between the RCOG or ACOG guidelines on birth after previous caesarean [3, 4, 20].

Another potential limitation of this internet survey is that the criteria used for the evaluation of website quality are not formally validated and the data scoring method is somewhat arbitrary. However, no consensus guideline for the evaluation of internet information exists. We therefore used criteria which have been cited as commonly used principles for this purpose [13–16]. In our analyses we have regarded all criteria as carrying equal weight in terms of importance. We cannot assume however that one website provides more reliable information than another, without first judging the individual importance of each criterion in clinical practice. This is especially important considering women are known to favour the delivery mode with least neonatal risk at the possible expense of increased maternal risk [21, 22]. Therefore websites may have scored highly despite failing to mention for example; the risk of increased neonatal mortality with VBAC, or increased risk of infant respiratory distress with ERCS.

Throughout this study, the RCOG guidelines and their supplementary patient information have been regarded as ‘gold standard’ [3, 8], with website information being scored against this. However it must be noted that the RCOG guidance has been based on best available evidence in the form of retrospective cohort studies and not randomised controlled trials. Additionally, the document was published in 2008, so there is potential for more recent research to supersede the document content. Despite this, it is the most comprehensive guidance available to date on which to base our findings, but results of future studies should prompt review of this internet survey.

It should also be noted that health information on the internet is a rapidly evolving field and the absolute results from this study will quickly become superseded by updated published guidance (e.g. NICE CG132 ‘Caesarean section’ [6]) and internet information (e.g. http://sdm.rightcare.nhs.uk/pda/birth-options-after-previous-caesarean/introduction/). In addition, there are websites available which provide good quality information on birth after caesarean that were missed using our chosen search method. Whilst acknowledging the unavoidability of such limitations, this study serves to provide a ‘snapshot’ of the nature and quality of internet information available to and accessed by women on this topic.

### Interpretation

These results are amongst the first regarding the reliability of internet information on birth after previous caesarean section. Despite any potential drawbacks, this study has demonstrated that internet information on this topic is highly variable in quality and in content. A large number of unregulated and unaccountable sources are providing potentially incomplete and somewhat misleading information. This is despite efforts to improve the quality of health information available on the internet [16]. On the other hand, some websites were deemed highly reliable and balanced when compared to official guidance. Considering that the general public are notably poor in interpreting the quality of internet information [17], obstetricians should be prepared to direct pregnant women towards appropriate advice on birth after previous caesarean section. This could be done when women present to the antenatal consultation, regardless of whether or not they already have a preconceived decision on delivery mode.

This study supports what is known from previous studies about health information on the internet, in that information can often be of poor quality and contain misleading content [19, 23–30]. However it should be noted that several websites accessed in this study actually scored favourably in relation to the ‘gold standard’ and appeared to convey high quality, reliable information (Table 3). In fact, the RCOG ‘gold standard’ patient information presented itself within the overall search results, in addition to NHS patient information leaflets which closely reflected RCOG guidance. It is reassuring that women have the opportunity to access the best quality information, but they may not necessarily have the ability to contextualise this amongst less reliable sources. It is of interest that websites from the UK featured more information supportive of repeat CS than those from other countries. This may relate to the majority of such websites being owned by national health services, who are accustomed to providing comprehensive patient information regarding treatment options which plays a role in reducing risk of litigation. As failed VBAC is a major source of litigation, it is possible that greater effort is made to ensure that information on its consequences, including scar rupture, and means of avoiding these through repeat CS, is widely available to patients.

The internet is known to be a substantial resource used by pregnant women for information throughout their pregnancy and is likely to be utilised by women researching birth after previous caesarean section [11, 12]. Our study confirms that a wealth of internet information exists on birth after previous caesarean section and has identified approximately three hundred different websites addressing this topic.

Interestingly none of our top web link results were ‘blog’ or ‘social networking’ style links even though these types of internet site are known to be accessed as a valuable means of support for pregnant women with previous caesarean section [22]. Perhaps this conveys that these ‘blog’ style websites lack generalisability since they are most often written, and even accessed, by highly educated, middle class American women [22]. Nevertheless ‘blog’ style websites have been found to strongly favour VBAC as the preferred birth mode, despite our results which show both modes of delivery after caesarean as equally supported on the most popular internet sites [22].

### Research and clinical implications

The findings of this study will inform care providers of the completeness of information and the most supported mode of birth to which patients may be exposed within their own home. This will enable delivery of advice regarding use of the internet for such purposes and may influence the development of future strategies to educate women on risks and benefits of modes of delivery after caesarean section via the internet.

Although this study has highlighted the availability of potentially poor quality, misleading internet information, many websites identified provided a high standard of information and balanced account of the options available. Combining this with incomplete advice often given by physicians themselves in comparison to official guidance [31, 32], then perhaps an area for further investigation is whether some internet healthcare information sources are actually more informative, accurate and balanced than that provided by healthcare professionals.

## Conclusion

Women searching for information on birth after previous caesarean section are exposed to incomplete internet information. Overall there was no significant difference in the amount of support given to method of delivery by the websites overall, but of the five most frequently returned websites, ERCS appears to be more significantly favoured. We found that commonly cited additional information regarding VBAC or ERCS on popular websites did not necessarily convey best available evidence. The findings of this study will inform health professionals counselling women regarding birth options after previous caesarean and may aid development of future interventions aimed at optimally informing women regarding birth options after caesarean section.
